# 30-day mortality in invasive candidiasis and candidemia in a multidisciplinary hospital in Moscow, Russia

**DOI:** 10.22034/cmm.2025.345248.1590

**Published:** 2025-04-14

**Authors:** Sergey S. Andreev, Polina O. Narusova, Anton A. Chernov, Alexander D. Dushkin, Olga D. Dukhanina, Rustam T. Iskhakov, Daria S. Fomina, Mariana A. Lysenko

**Affiliations:** 1 Clinical City Hospital No. 52, Ministry of Healthcare of Moscow, Moscow, Russia; 2 First Sechenov Moscow State Medical University (Sechenov University), Moscow, Russia; 3 Russian Medical Academy of Continuous Professional Education, Moscow, Russia; 4 Pirogov Russian National Research Medical University, Moscow, Russia

**Keywords:** Antifungal therapy, Candidemia, Healthcare-associated infections, Invasive candidiasis, Mortality

## Abstract

**Background and Purpose:**

One of the most severe mycotic infections caused by *Candida* spp. is invasive candidiasis. According to the literature, among all healthcare- associated infections, it has the highest mortality rate. This study aimed to assess 30-day and overall mortality in invasive candidiasis and candidemia patients depending on the antifungal therapy (AFT) regimens.

**Materials and Methods:**

This single-center retrospective study of 30-day survival was conducted at Clinical City Hospital No. 52, Moscow Healthcare Department in Moscow, Russia. The participants were 169 patients aged 19-94 years who had verified invasive candidiasis with candidemia during hospitalization in 2020–2023. This study included patients with *Candida* spp. isolated from blood culture using matrix-assisted laser desorption/ionization with time-of-flight mass spectrometry, and proven invasive candidiasis according to EORTC/MSG criteria. Patient survival analysis was performed using the Kaplan-Meier method, which is a nonparametric approach for estimating time- to-event. Risk of death was compared between the group of patients receiving AFT after pathogen verification and the group of patients receiving AFT before and after blood culture results.

**Results:**

Based on the findings, the likelihood of death was lower in the group of patients who received AFT both after and before blood culture results compared to the group of patients who received it after verification of the diagnosis. By day 50 of hospitalization, the risks of death were comparable between the two groups. However, when analyzing the overall mortality, the odds of death in patients with AFT before and after receiving blood culture results were 2.56 times higher (OR=0.391; 95% CI: 0.177–0.865; *p*=0.019) compared with patients to whom antifungal therapy was prescribed only after blood culture results.

**Conclusion:**

This study provided the first data regarding the assessment of 30-day mortality and risk factors for death. Risk of 30-day mortality was lower in the group of patients receiving AFT both before and after the blood culture, but overall mortality in this group was higher, compared to patients who received AFT after the blood culture.

## Introduction

One of the most severe mycotic infections caused by *Candida* spp. is invasive candidiasis (IC), and blood culture is the gold standard for its diagnosis [ 1 ]. There are at least 15 different strains of *Candida* that can potentially cause IC, but 95% of IC cases are caused by the following six strains: *Candida albicans*, *Candida glabrata*, *Candida tropicalis*, *Candida parapsilosis*, *Candida krusei*, and *Candida auris* [ 2 , 3 ].

According to the literature, IC is one of the most common healthcare-associated infections (HAIs) and is the most common of the mycotic HAIs [ 2 ]. A study performed in 2018 in the USA found that candidemia, the most common form of IC [ 4 ], is the fourth most common HAI and the second most common healthcare- associated bloodstream infection [ 5 ]. Moreover, patients in intensive care units (ICUs) and immunosuppressed patients are at the highest risk of developing IC. [ 2 ]. In addition, injection drug users are also at risk of developing IC [ 6 ].

Risk factors for invasive candidiasis can be divided into three groups, namely concomitant diseases, drug therapy, and other factors associated with the provision of health care. [ 7 ]. Concomitant diseases and conditions that increase the risk of developing IC include: oncological and oncohematological diseases, diabetes mellitus, conditions after solid organ and hematopoietic stem cell transplantation, graft-versus-host disease, liver and kidney failure, pancreatic necrosis, neutropenia, and severe mucositis.

Drugs that increase the risk of developing IC include glucocorticosteroids (more than 0.3 mg/kg/day for more than 3 weeks), antitumor drugs, immunosuppressants, and broad-spectrum antibiotics [ 8 , 9 ].

In-hospital IC risk factors are a long ICU stay, mechanical ventilation, major surgeries (especially those involving disruption of the intestinal tube), total parenteral nutrition, repeated blood transfusions, intestinal anastomosis failure, the presence of a central venous catheter, a left ventricular assist device, and renal replacement therapy.

According to the literature, among all HAIs, IC has the highest mortality rate. On the 30th day of hospitalization after the diagnosis verification, the mortality rate reaches 40–55% [ 10 , 11 ]. In a study conducted in 2021 on 207 Chinese patients with cancer and IC, the 30-day mortality rate reached 28% [ 12 ]. Similar results were obtained in a 2019 retrospective study conducted in Japan on 289 patients with IC [ 13 ]. However, in a 2020 retrospective study conducted in Germany which included data on 391 patients, the 30-day mortality rate was 47%, which is significantly higher, compared to the above-mentioned studies [ 14 ]. Conflicting data on hospital survival of IC patients determined the aim of the present study which was to evaluate 30-day mortality in patients with invasive candidiasis and candidemia in a multidisciplinary hospital in Moscow, Russia.

## Materials and Methods

A single-center retrospective study of 30-day survival was conducted at the Clinical City Hospital No. 52, Moscow Healthcare Department on 169 patients aged 19-94 years who had verified IC with candidemia (B37.7 according to ICD-10) during hospitalization in 2020–2023.

The study included patients with *Candida* spp. isolated from blood culture using matrix-assisted laser desorption/ionization with time-of-flight mass spectrometry (MALDI-TOF MS, Bruker), and proven invasive candidiasis according to the European Organization for Research and Treatment of Cancer/Invasive Fungal Infections Cooperative Group/Mycoses Study Group (EORTC/MSG) criteria. Overall mortality was analyzed within 30 days from the moment *Candida* spp. was isolated from blood culture. It should be mentioned that the risk of death was assessed in 10-day increments.

Patient survival analysis was performed using the Kaplan-Meier method, which is a nonparametric approach for the estimation of time-to-event. The survival function was defined as the probability that the time until an event occurs will be greater than a certain value. Statistical comparison of Kaplan Meier survival curves was performed using the log-rank test.

The risk of death was further compared between the group of patients receiving antifungal therapy (AFT) after pathogen verification (Group I) and the group of patients receiving AFT before and after blood culture results (Group II – comparison group).

This study was approved by the local ethics committee of the State Budgetary Healthcare Institution “City Clinical Hospital No. 52 of the Moscow Department of Health” (protocol No. 07/0724 dated July 31, 2024) and was conducted in accordance with Russian regulations and the Declaration of Helsinki of the World Medical Association.

## Results

**Figure 1:**
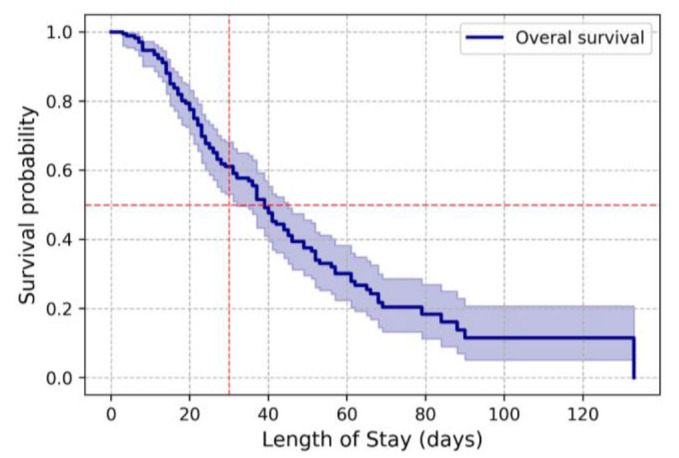
Overall survival of patients with invasive candidiasis and candidemia.

In patients of the general cohort (n=169), the risks of death on days 20, 30, and 40 were 22.5% (95% CI: 16.9-29.7), 38.9% (95% CI: 31. 8-47.0), and 52.4% (95% CI: 44.6-60.7), respectively. Median survival was 39 days (95% CI: 32-45) (Figure 1, Table 1).

**Table 1:** Data for Kaplan-Meier survival analysis of hospital length of stay.

Length of Stay (days)	0	10	20	30	40	50	60	70	80	90	100	110	120
Observations	169	158	121	89	59	42	27	13	9	5	4	4	3
Censored	0	2	11	18	30	35	42	48	51	52	53	53	54
Events	0	9	37	62	80	92	100	108	109	112	112	112	112

**Figure 2:**
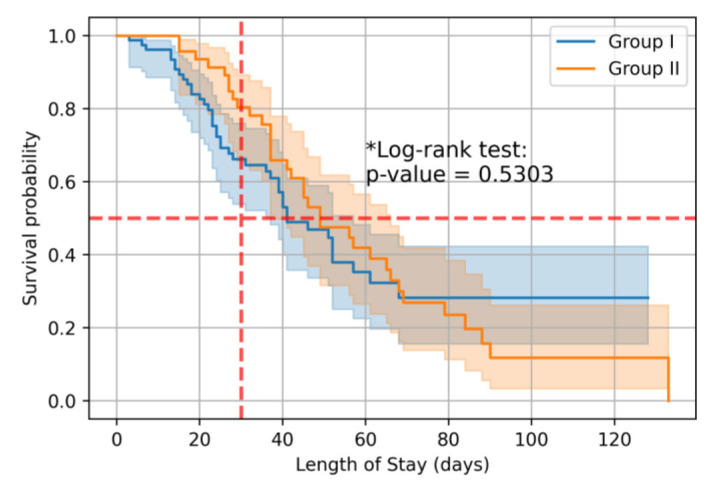
Overall survival of patients with invasive candidiasis and candidemia depending on investigation groups.

Only data from patients who received AFT (n=124) were included in further analysis. In 45 patients, the absence of AFT was due to the prolonged time required to obtain blood culture results. When analyzing 30-day mortality in patients with IС, the likelihood of death was lower in the group of patients who received AFT both after and before blood culture results compared to the group of patients in whom AFT was prescribed after verification of the diagnosis (Table 2, Figure 2). Median survival for patients receiving AFT before and after blood culture results was 49 days (95% CI: 37-66 days), versus 41 days (95% CI: 37-57 days) for patients receiving AFT only after blood culture results (*p*=0.5303). By day 50 of hospitalization, the risks of death were comparable between groups (Table 2 and 3). However, when analyzing overall mortality, the odds of death in patients with AFT before and after receiving blood culture results were 2.56 times higher (OR = 0.391; 95% CI: 0.177–0.865; p = 0.019) compared with patients to whom AFT was prescribed only after blood culture results (Table 4).

**Table 2:** Risk of death based on the investigation groups.

Length of Stay (days)	Cumulative mortality risk in percentage (95% CI)	
	Group I	Group II
20	17.4% (10.5-28.1)	6.5% (2.2-18.9)
30	33.8% (24.1-46.2)	19.6% (10.7-34.3)
40	46.9% (35.4-60.1)	34.1% (22.1-50.2)
50	53.2% (41.0-66.4)	52.5% (38.2-68.5)
60	64.8% (51.8-77.5)	58.1% (43.4-73.6)
70	71.8% (57.7-84.4)	73.1% (58.2-86.1)
80	71.8% (57.7-84.4)	76.4% (61.6-8.8)
90	71.8% (57.7-84.4)	88.2% (73.8-96.7)

CI: confidencel interval

**Table 3:** Data for Kaplan-Meier survival analysis of hospital length of stay depending on investigation groups.

Length of Stay (days)	Antifungal therapy after blood culture (Group I)												
	0	10	20	30	40	50	60	70	80	90	100	110	120
Observations	78	73	58	42	26	21	12	5	2	2	1	1	1
Censored	0	2	7	12	21	23	27	32	35	35	36	36	36
Events	0	3	13	24	31	34	39	41	41	41	41	41	41
**Antifungal therapy before and after blood culture (Group II)**													
Observations	46	46	43	35	27	17	14	8	7	3	3	3	2
Censored	0	0	0	2	4	7	8	9	9	10	10	10	11
Events	0	0	3	9	15	22	24	29	30	33	33	33	33

**Table 4:** Hospitalization outcomes based on investigation groups.

Outcomes	Group I	Group II	p value
Favorable	37/47,4	12/26,1	0,019*
Unfavorable	41/52,6	34/73,9	

## Discussion

In a 2020 retrospective study of 163 patients with IC, the overall 30-day mortality rate was 40.5%. The results revealed that the risk of death at 30 days was significantly higher in patients on hemodialysis (n=27 (69.2%) vs. n = 12 (30.8%), *p*<0.00) and in patients with concomitant bacteremia (n=20 (57.1%) vs. n = 15 (42.9%), *p*=0.024). The study also assessed the effect of various antifungal drugs on 30-day mortality and found no statistically significant differences between the drugs [ 15 ].

In a 2020 retrospective study, 28-day mortality in 391 patients with IC was 47%, increasing to 60% at 180 days. Factors influencing the risk of death at 180 days were age (RR 1.02 [95% CI 1.01-1.03]), cirrhosis (RR 1.54 95% CI 1.07-2.20]), septic shock (RR 2.41 [95% CI 1.73-3.37]), high Sequential Organ Failure Assessment (SOFA) scores (OR 1.12 [95% CI 1.07- 1.17]), the ICU stay of the patient at the time of receiving blood culture results. When prescribing adequate (RR 0.36 [95% CI 0.24-0.52]) or inadequate AFT (RR 0.31 [95% CI 0.16-0.62]) the risk of death was lower, compared to the group of patients without therapy [ 14 ].

A study conducted in 2017 included 102 patients with severe invasive candidiasis. Their overall 30-day mortality rate was 68.6%. Independent risk factors of death on day 30 were sepsis (*p*=0.001, OR 7.7, 95% CI 2.4-24.7) and Charlson comorbidity score of more than three (*p*=0.022, OR 3.5, 95% CI 1.2-10.2) [ 16 ].

In a 2019 multicentric study of 289 patients with IC, the overall 30-day mortality rate was 27.7%. According to the results of logistic regression, risk factors for 30-day mortality were age of more than 65 years and a SOFA score greater than or equal to 6. Factors that positively influenced the prognosis were repeated blood cultures and empirical AFT with fluconazole [ 13 ].

In 2018, a study was carried out on 121 patients with IC, and the 30-day mortality rate was 33%. The study found that risk factors for death included *C. albicans* as a pathogen, absence of AFT, advanced age, pulmonary disease, and mechanical ventilation. Factors that positively influenced the prognosis were *C. parapsilosis* as a pathogen, removal of the central venous catheter, and stay in the surgical department [ 17 ].

A study performed in 2018 evaluated different AFT regimens in 841 patients with candidemia. Empirical therapy was associated with a high risk of *Candida* spp. infection (except *C. albicans*). Empirical echinocandin treatment was associated with more patients receiving adequate therapy and fewer 14-day therapy durations. To date, studies evaluating the effect of AFT on the risk of 30-day mortality have compared the presence and absence of AFT, the effects of different antifungals, empirical fluconazole therapy versus empirical therapy with other drugs, and empirical therapy with echinocandins and azoles. No data are available on studies that compared the risk of 30-day mortality of patients in whom AFT was initiated before blood cultures and continued thereafter with those in whom AFT was initiated after the blood culture.

The present study found that the risk of 30-day mortality of patients in whom AFT was initiated before blood cultures and continued thereafter was significantly higher, compared to those in whom AFT was initiated after the blood culture. This may be due to the fact that AFT administration prior to blood culture reduces the overall incidence of candidemia in high/very high-risk patients, but when IC develops, it will be caused by more resistant *Candida* spp. (primarily C. auris).

## Conclusion

This study assessed the 30-day mortality rate and risk factors for death and provided the first data in this regard. Accordingly, the risk of 30-day mortality was lower in the group of patients receiving AFT both before and after the blood culture; however, the overall mortality rate was higher in this group, compared to patients in whom AFT was initiated after the blood culture. Further research is needed to determine the AFT regimens in patients at high risk of developing invasive candidiasis.
